# A conceptual framework for the public health monitoring of substance-related harms

**DOI:** 10.24095/hpcdp.45.2.02

**Published:** 2025-02

**Authors:** Heather M. Orpana, Aganeta Enns, Megan Striha, Diana George, Abban Yusuf, Stephanie L. Hughes, Le Li, Laura H. Thompson

**Affiliations:** 1 Centre for Surveillance and Applied Research, Health Promotion and Chronic Disease Prevention Branch, Public Health Agency of Canada, Ottawa, Ontario, Canada; 2 School of Psychology, University of Ottawa, Ottawa, Ontario, Canada

**Keywords:** substance-related harms, opioids, overdose, substance use, surveillance, monitoring

## Abstract

The drug toxicity crisis in Canada and elsewhere has increased the need for timely and relevant data to inform policies and programs aimed at mitigating substance-related harms. While a number of monitoring systems addressing specific components of substance use and related harms in Canada exist, they are not guided by an overarching conceptual framework. This evidence-informed policy brief describes the development of a conceptual framework for the public health monitoring of substance-related harms. The resulting framework includes four primary topic areas (risk and protective factors, substance use, health supporting systems and substance-related harms and benefits); four cross-cutting topic areas (life course, equity, substance use stigma and mental and physical health and illness); and two overarching considerations (respectful use of data and engagement). This framework can be used to organize existing activities and to identify data and monitoring gaps for further development.

**Highlights**

- Comprehensive and timely data are essential to inform efforts to address the drug toxicity crisis and other substance-related harms. Currently, no overarching conceptual framework guides the monitoring of substance-related harms in Canada.
- A comprehensive conceptual framework for the public health monitoring of substance-related harms was developed to help guide related efforts.
- The conceptual framework described here includes four primary topic areas, four cross-cutting topic areas and two overarching considerations.

## Introduction

The opioid and other drug toxicity crisis was identified in 2016 as a significant national public health concern in Canada,^1^,^2^ leading to the creation of a federal, provincial and territorial Special Advisory Committee (SAC) on Toxic Drug Poisonings (initially called the SAC on the Epidemic of Opioid Overdoses) in December 2016.^3^ The overprescription of opioids in health care settings in past decades, increasing the risk of opioid use disorder for some patients,^2^ set the stage for a higher level of harms once synthetic opioids such as fentanyl and carfentanil became more available in the unregulated drug market.^1^

Tragically, the drug toxicity crisis has worsened since the onset of the COVID-19 pandemic.^3^,^4^ At the same time, harms from other substances, such as alcohol, continue to have an important impact on population health. One component of Canada’s response to the drug toxicity crisis continues to be improved data and monitoring.^5^ “Surveillance” is considered a foundational activity of public health, and is defined as “the ongoing, systematic collection, analysis and interpretation of health data, essential to the planning, implementation and evaluation of public health practice, closely integrated with the dissemination of these data to those who need to know and linked to prevention and control.”^6^^,p.3^ While the term “surveillance” has a long history in public health, it is also used in other sectors, such as law enforcement and private security. Because of this, it may cause discomfort and have negative associations for some people and communities.^7^,^8^ Thus, we have chosen to use the word “monitoring” where possible.

Throughout this paper, the terms “drugs” and “substances” are used interchangeably to refer to psychoactive substances that may be regulated (such as alcohol and cannabis), substances from the unregulated drug supply or psychoactive pharmaceutical drugs not prescribed to the individual or not used as directed by a health professional. 

In 2017, the Public Health Agency of Canada (PHAC) led the development of a national surveillance system for apparent opioid related deaths (AORDs), with the goal of generating a timely, national picture of the public health impact of opioids in Canada.^3^ This system evolved to include other types of harms including hospitalizations, emergency department visits and emergency medical services contacts, and other substances, namely stimulants.^5^ While opioids, and subsequently stimulants, have been at the forefront of attention focussed on substance-related harms due to the drug toxicity crisis, there is a broad recognition that deaths, hospitalizations and emergency medical services contact for poisonings (overdoses) related to opioids and stimulants are only one subset of the information needed for a comprehensive approach to address substance-related harms from a public health perspective.^9^

Canada has a number of data and monitoring programs related to substances. For example, Health Canada supports the Canadian Alcohol and Drugs Survey, which collects data on a biennial basis about substance use among Canadian adults.^10^ Data on substance use is included in many cycles of the Canadian Community Health Survey,^11^ including targeted modules on special topics, such as the use of pain relieving medications that include opioids.^12^ The Canadian Institute for Health Information collects, analyzes and disseminates data on hospital stays from harms due to substance use, among other substance-related harms indicators.^13^ The Canadian Substance Use Costs and Harms project, a collaboration between the Canadian Centre on Substance Use and Addiction and the Canadian Institute for Substance Use Research, synthesizes data about harms and costs associated with substance use in Canada.^14^ However, currently no overarching conceptual framework has been presented to guide, organize and integrate federal monitoring activities on substance-related harms, which may limit effective organization, collection, analysis and dissemination of data that can be used to inform prevention and promotion efforts.

The purpose of this evidence informed policy brief is to describe and document the development process and outcome of a conceptual framework for the public health monitoring of substance-related harms. A conceptual framework can be described as “a set of linked concepts and propositions designed to draw attention to what is important regarding a phenomenon of interest.”^15^^,p.631^ Conceptual frameworks can be used to clarify, focus, describe and organize.^16^ While the outcome of this process is not meant to be prescriptive, it can be used to guide thinking about the development of future initiatives to bolster monitoring efforts in the area of substance-related harms.

## Framework development

In late 2019, we conducted a literature review to identify existing conceptual frameworks for the monitoring of substance-related harms. While no conceptual frameworks were identified that focussed specifically on substance-related harms, a number of existing monitoring systems in Canada, the United States and other countries focussed on substance use and/or a limited number of related health outcomes, such as alcohol and drug use^10^,^17^,^18^ or opioid- and stimulant-related harms.^5^ In November 2023, we refreshed our search to identify any new conceptual frameworks focussed specifically on the monitoring of substance-related harms. At that time, we were still unable to identify any such conceptual frameworks and continued to observe that data or monitoring systems tended to focus on substance use or on a limited range of substance-related harms without guiding conceptual frameworks.

While no standard process has been articulated for the development of conceptual frameworks for public health monitoring, generally these frameworks are developed using initial literature review followed by rounds of iterative feedback from relevant stakeholder groups.^19^,^20^ Using the results of the 2019 literature review and drawing from existing monitoring frameworks in health promotion and chronic disease prevention at PHAC^19^,^21^ as well as the Chief Public Health Officer’s 2018 report, *Preventing Problematic Substance Use in Youth*,^22^ a baseline conceptual framework was developed. The initial visual framework aligned with the broad components of existing surveillance frameworks at PHAC (such as the Positive Mental Health Surveillance Indicator Framework^19^ and the Suicide Surveillance Indicator Framework.^21^ These frameworks identify outcomes of interest, as well as risk and protective factors at four socioecological levels; acknowledge that included constructs may vary across the life course; and emphasize that surveillance must be able to capture priority populations. 

**Figure 1:**
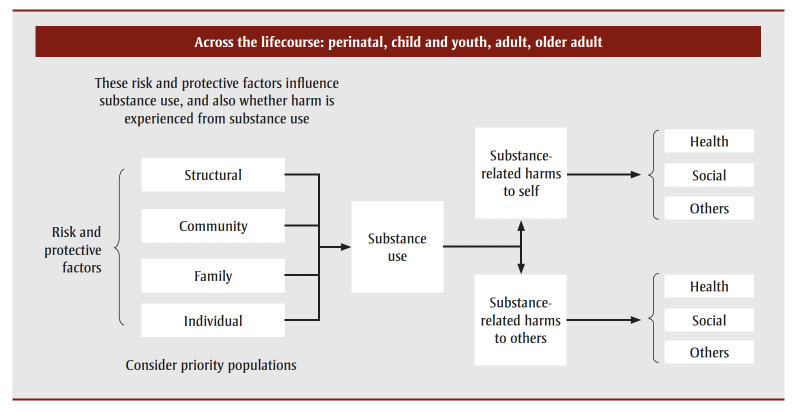
Initial conceptual framework for the surveillance of substance-related harms

The initial framework presented substance use as distinct from substance-related harms, and identified that harms extend beyond harms to the health of the person using substances. This framework was drafted by members of the development team, based on interpreting the key findings from the literature review, establishing the scope of the framework, identifying concepts and considerations and drafting the initial visual conceptual framework to illustrate the relationships between the constructs (Figure 1). 

This initial conceptual framework was presented in several iterative rounds of engagement with key internal stakeholders at PHAC and Health Canada, as well as people with lived and living experience of substance use (PWLLE). Internal stakeholders were identified as employees of groups within PHAC or Health Canada with a responsibility for or significant interest in substance-related harms. This included groups with responsibilities for policy, programs, surveillance and applied research. PWLLE were engaged through Health Canada’s People with Lived and Living Experience Council. Engagement sessions were conducted online through Teams or Zoom, using the services of a professional facilitator with extensive experience conducting engagement sessions. The online visual collaboration tool, Mural, was used to facilitate visualization and collect feedback about the proposed conceptual framework during these sessions. These sessions were undertaken as part of the usual course of actions in developing approaches to federal monitoring activities.

In advance of engagement sessions, participants were provided with the most recent version of the conceptual framework for review and were asked to arrive prepared to share their thoughts and discuss their feedback. Engagement sessions lasted from 30 minutes to up to 2.5 hours, with a longer duration for larger groups. All sessions began with a description of the context and history of framework development and a walk-through of the most recent iteration of the framework. Participants in larger engagement sessions were then given an opportunity to discuss feedback in small break-out groups prior to a full group discussion, which took place for all engagement sessions. During these engagement sessions, feedback and discussion points were documented and participants were invited to follow up afterwards with any additional feedback or questions by email.

Primary topic areas can be considered content areas for monitoring activities, while the cross-cutting topic areas can be considered as lenses through which primary topic areas can be viewed. Feedback from engagement sessions resulted in changes to primary and cross-cutting topic areas and overarching considerations. Additionally, we documented information that could inform the scope of a topic area or what type of information would be important within that topic area (data not shown). Additional topic areas that were incorporated based on feedback from engagement sessions included “substance-related benefits” (in addition to harms), “stigma,” “mental and physical health and illness” and “health-supporting systems.” 

**Figure 2:**
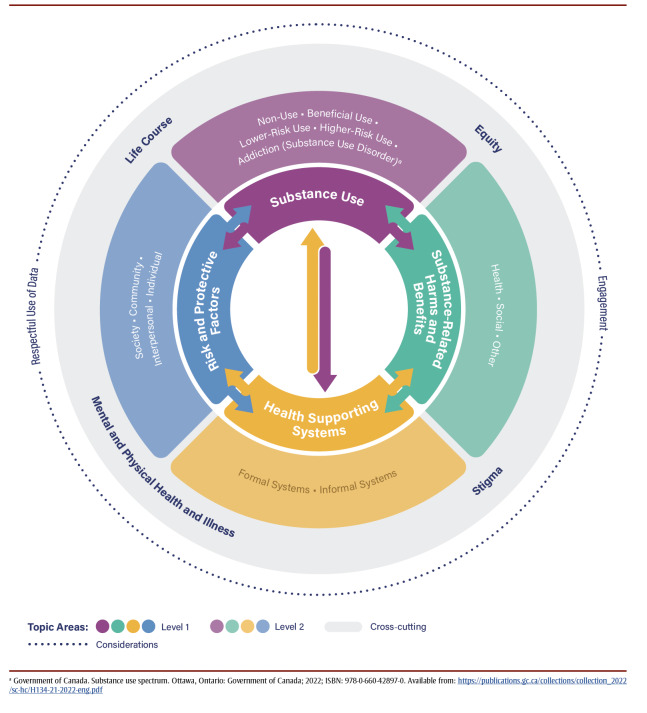
Conceptual framework for the public health monitoring of substance-related harms

The concept that was originally termed “priority populations” was renamed as a cross-cutting topic area of “equity.” Additionally, two overarching considerations were added, as they were identified as foundational to monitoring work in this area: “engagement” with PWLLE, and “respectful use of data.” The resulting framework, shown in Figure 2, includes four primary topic areas, four cross-cutting topic areas and two overarching considerations. Each of these is described in detail below.

***Primary topic areas***

**Risk and protective factors**

Risk and protective factors (a Level 1 concept; see Figure 2) can occur at four socioecological levels (a Level 2 concept)— individual, interpersonal, community or societal.^23^ Individual factors are unique to the person, and are related to biological, behavioural, demographic or socioeconomic characteristics.^24^ Interpersonal factors are related to relationships with other persons. Community factors are related to groups with a shared identity or geography within the social or physical environment.^25^ Societal factors are related to social, political, legal and economic structures, policies and systems, and are broadly embedded throughout society, through both formal and informal mechanisms. 

**Substance use**

The substance use spectrum represents the use of psychoactive substances, which includes substances that “when taken in or administered into one’s system, affect mental processes, e.g. cognition or affect,”^26^ such as opioids, stimulants, cannabis or alcohol. Substance use (a Level 1 concept) occurs on a spectrum, which can range from non-use through beneficial use, lower-risk use and higher-risk use to addiction/dependence (substance use disorder; Level 2 concepts).^27^,*

Additional characteristics of substance use could be considered, depending on the contexts and use of the monitoring system, such as length of time the substance has been used, frequency of use, mechanism of consumption, source of substance and contexts within which the substance is used, among others. While not specifically substance use, the substance supply system is an important determinant of substance use and related harms, and should be considered in conjunction with the substance use spectrum. 

**Health supporting systems**

Health supporting systems (a Level 1 concept) include both formal and informal systems (Level 2 concepts). Formal health supporting systems refer to the organized and structured network of health services and facilities that operate within a legal and professional framework, such as hospitals, primary health care and psychological services. Informal health supporting systems, on the other hand, encompass a wide range of health-related support that exists outside the formal health care infrastructure. This may include peer-based support, family care and care provided by volunteers, including services for people who use substances. The language of this primary topic area is specifically worded to highlight that it is not only the formal health care system that is important for data and monitoring in this context. Other formal systems, such as the education, social services, housing or legal systems, can support (or fail to support) health. Attributes of formal health supporting systems may be characterized by accessibility, approachability, acceptability, availability and accommodation, affordability and appropriateness.^28^ These attributes could be incorporated into future monitoring efforts. 

**Substance-related harms and benefits**

Substance-related harms (a Level 1 concept) are negative effects that result from the use of psychoactive substances. In the present framework, harms are categorized as “health,” “social” and “other” (Level 2 concepts). Harms can be acute (short lasting) or chronic (long lasting), proximal (close in time) or distal (far in time) to the substance use, and may be experienced by oneself or others. An example of an acute, proximal harm to oneself would be a drug poisoning (overdose), while a chronic distal harm could be the long-term effects of alcohol consumption such as alcohol-related liver cirrhosis. Someone injured in a motor vehicle accident in which the other driver was intoxicated is an example of a substance-related harm to another person. 

Health harms can include negative effects on physical or mental health, or both. Social harms can include problems with one’s interpersonal relationships or role functioning (i.e. the ability of an individual to perform the functions of developmentally appropriate roles such as student, parent or worker). Other harms may be economic (e.g. loss of one’s employment income) or legal (e.g. interaction with the justice system).

While substance-related harms are often the focus of public health monitoring, it is important to keep in mind that there are also real or perceived benefits to substance use, which is often the reason people continue using a substance in the face of harms. Benefits that may result from the use of psychoactive substances may include health or social benefits, such as reduced pain or greater sociability.^27^

***Cross-cutting topic areas***

Four cross-cutting topic areas were identified as applicable across the four primary topic areas within the framework.

**Life course**

The life course cross-cutting topic area reflects that components of the framework may be relevant at, and vary across, multiple developmental stages.^29^ The life course includes biological age and developmental stage, and also encompasses life events and transitions (e.g. pregnancy and lactation, parenthood, marriage, divorce, death of a close family member or friend, changes at work or to employment status, retirement). Certain risk and protective factors, harms and benefits may have a greater impact at a particular age and may also change over the life course. In addition, the life course must be considered within its historical, cultural and socioeconomic context, which can vary through developmental stages. 

**Equity**

The concept of equity underpins the structure and components of the conceptual framework.^30^ Substance-related harms and associated risk and protective factors, substance use behaviour, stigma and service access and provision differ between demographic and socioeconomic groups due to the impacts of cultural and structural systems that assign value and grant opportunities and privileges based on these characteristics. Data about who is most affected provides the foundation for targeted policies and programs.^31^ Approaches to analyzing health inequities, such as Sex- and Gender Based Analysis Plus (SGBA+),^32^ should be integrated into all substance-related harms monitoring activities, with consideration of the impact of broader systems, policies and structures that shape these inequities. 

**Substance use stigma**

Substance use stigma “is negative attitudes, beliefs or behaviours about or towards a group of people because of their situation in life. It includes discrimination, prejudice, judgment and stereotypes, which can isolate people who use drugs.”^33^ Stigma can take several forms: self-stigma, in which someone internalizes within themselves negative attitudes about people who use substances; social stigma, which is others holding negative attitudes and behaviours towards people who use drugs; and structural stigma, which occurs when policies and practices reduce access to services by people who use drugs.^34^ All of these types of stigma may lead to a greater chance of negative outcomes for people who use drugs.^35^

**Mental and physical health and illness**

Mental and physical health and illness were identified as a fourth cross-cutting topic area. While harms to health is a subtopic area under “Substance-related harms and benefits,” based on feedback from engagement sessions, this additional cross-cutting topic area was added.^36^-^38^ For example, pain resulting from acute or chronic health conditions may influence substance use as well as treatment received for substance use disorder.^36^ Mental disorders and substance use disorders often co-occur;^37^,^38^ their co-occurrence may impact access to services for both conditions.^39^ A particularly novel area may be a focus on positive mental and physical health in this cross-cutting topic area. While much of the discourse related to substance use focusses on harms (or the prevention thereof), focussing on positive health may yield previously overlooked benefits. This is consistent with a substance use health perspective, which “suggests that substance use not be considered in isolation but rather as an overall component of health and well-being.”40

***Overarching considerations***

Finally, the proposed framework also includes two overarching considerations: engagement with PWLLE and respectful use of data. Engagement with PWLLE is foundational to any evidence development in the area of substance use, and its related harms and benefits.^41^ This stems from a foundational principle that work done in the interest of a community should actively engage that community, and that communities are experts through their lived experience.^42^

Additionally, the consideration of respectful use of data encompasses the principle that data should be collected and used in ways that will neither further stigmatize nor harm the community of people who use drugs or who are affected by substance-related harms. This is consistent with the concepts of cultural responsiveness and accessibility in order to avoid perpetuating systematic discrimination and harms.^43^ Consistent with this is the identification and application of appropriate guiding frameworks, such as Ownership, Control, Access and Possession (OCAP) for Indigenous data^44^ and Engagement, Governance, Access and Protection (EGAP) for race-based data from Black communities.^45^

*The term “addiction/dependence” is used in the original spectrum; however, the preferred term is “substance use disorder.”

## Discussion

This paper presents the process we undertook to develop a conceptual framework for public health monitoring of substance-related harms, a critical component in addressing the ongoing drug toxicity crisis and broader challenges related to substance use in Canada,^46^ such as those caused by alcohol. The development of this framework is timely and necessary, considering the continuing public health concerns related to substance use and its associated harms, which have been exacerbated by the COVID-19 pandemic.^4^

The framework’s emphasis on a multifaceted approach, integrating risk and protective factors across four socioecological levels (individual, interpersonal, community and societal), aligns with other monitoring frameworks in chronic disease and health promotion at PHAC. This approach acknowledges the complexity of substance use and its impacts, which extend beyond individual behaviour to encompass interpersonal, community and societal influences. The framework’s inclusion of a spectrum of substance use behaviours, from non-use through beneficial use, lower-risk use and higher-risk use to addiction/dependence (substance use disorder), allows for a more nuanced understanding of substance-related harms as well as benefits. This is crucial for developing universal interventions to shift the population distribution of substance-related harms broadly,^47^ as well as targeted interventions that are sensitive to the varied experiences and needs of different population segments.^31^

The integration of health supporting systems into the framework underscores the vital role of both formal and informal systems in supporting the health of people who use substances and in mitigating substance-related harms. Considering aspects that affect access to services may be particularly important, given the disparities in health outcomes among different demographic groups. Disparities in health outcomes give rise to the need for the cross-cutting topic area of equity, and highlight the importance of ensuring that monitoring efforts are inclusive and address the needs of equity-seeking populations. Cross-cutting topic areas—the life course perspective, equity, stigma and mental and physical health and illness—highlight the dynamic nature of substance use and its impacts over an individual’s lifespan, the importance of addressing systemic inequities and stigma, and the interplay between substance use, mental and physical health conditions and experienced harms and benefits.

***Strengths and limitations***

The iterative development process of the framework, involving engagement with a diverse range of stakeholders from the organizations that would use this framework to guide their efforts, and including PWLLE, adds to its robustness and relevance. This approach not only ensures that the framework is grounded in real-world experiences and needs but also should enhance buy-in and uptake across the participating organizations.

The development process of this conceptual framework, while comprehensive, has certain limitations. To begin with, the sequence of engaging a wide range of groups might have influenced the final framework: a different engagement order could have yielded an alternate framework structure. 

Another limitation is the restricted range of stakeholders involved in the consultation. Input was sought from groups within the federal health portfolio as well as PWLLE of substance use. This selective approach may have overlooked diverse perspectives from stakeholders in other sectors or government levels, which could have led to a different framework structure. An example of this is that the substance supply market did not emerge as a distinct topic area. This may be because the concept of the source of substances is often coupled with measurement of substance use and substance-related harms in the public health sphere. Finally, it was not possible to include all feedback directly into the framework. Ongoing engagement with users of monitoring data is essential if these primary and cross-cutting topic areas are used to inform concrete monitoring activities, such as identifying indicators and measures. 

This framework can be used by organizations to examine existing data collection activities and monitoring systems, including whether current indicators and measures may align with the primary and cross-cutting topic areas. We anticipate that this framework will be a useful tool for other organizations and levels of government to inform their own monitoring frameworks and we expect that it will be further developed in an iterative manner. Additionally, it provides a structure with which to identify gaps in these topic areas, which can be filled through the identification of relevant indicators and measures. Cross-cutting topic areas should be considered across the primary topic areas; e.g. stigma should be considered across risk and protective factors, substance use, health supporting systems and substance-related harms and benefits.

The considerations of respectful use of data and engagement with PWLLE should be taken into account at all subsequent steps of application of this conceptual framework into practical activities. Identifying these considerations within this framework is a step in the right direction, but ongoing commitment and resources to ensure these principles are upheld in practice will be required. Finally, we note that monitoring activities almost exclusively focus on the collection of data that can be summarized quantitatively—prevalences, incidences, counts and proportions, for example. However, given the rich input provided, particularly by PWLLE of substance use, those responsible for monitoring systems may find qualitative data helpful in contextualizing more traditional monitoring data sources.

## Conclusion

This conceptual framework represents an additional step towards a more comprehensive approach to the public health monitoring of substance-related harms. It is a multidimensional framework that can guide future initiatives. However, its successful application will depend on continued collaboration among stakeholders, the identification of existing activities and their alignment within this framework, the identification of gap areas and potential mechanisms to fill them and a commitment to meaningful engagement of communities affected by substance use.

## Acknowledgements

We deeply appreciate and are grateful for the input provided by Jill Harnum, Nat Kaminski, Ashley Smoke, Matthew Bonn and Petra Schulz. We also acknowledge the assistance of Tanner Maw with manuscript preparation and Emilene Reisdorfer for her work on the initial literature review.

## Conflicts of interest

None.

## Authors’ contributions and statement

HMO: conceptualization, methodology, investigation, project administration, visualization writing—original draft.

AE: investigation, validation, writing—review and editing.

MS: methodology, investigation, visualization, writing—review and editing.

DG: conceptualization, investigation, visualization, project administration, writing—review and editing.

AY: investigation, writing—original draft, writing—review and editing.

SLH: investigation, writing—review and editing.

LL: investigation, writing—review and editing.

LHT: methodology, investigation, visualization, writing—original draft, writing—review and editing.

The content and views expressed in this article are those of the authors and do not necessarily reflect those of the Government of Canada.
